# Emotion dysregulation in children and adolescents with externalizing disorders: a systematic review and meta-analysis

**DOI:** 10.1007/s00787-026-03090-8

**Published:** 2026-06-16

**Authors:** Urdur Njardvik, Gro Janne Wergeland, Krister W. Fjermestad, Juliette M. Liber, Caitlin M. Prentice, Eili N. Riise, Anne Mari Syversen, Lars-Göran Öst

**Affiliations:** 1 Department of Psychology, University of Iceland, Saemundargata 2, Reykjavik, IS-102 Iceland; 2 Department of Child and Adolescent Psychiatry, Division of Psychiatry, Haukeland University Hospital, Bergen, Norway; 3 Department of Clinical Medicine, Faculty of Medicine, University of Bergen, Bergen, Norway; 4 Department of Psychology, University of Oslo, Oslo, Norway; 5 Department of Developmental Psychology, University of Amsterdam, Amsterdam, Netherlands; 6 Department of Child and Adolescent Psychiatry, District General Hospital of Førde, Førde, Norway; 7 Department of Psychology, Stockholm University, Stockholm, Sweden

**Keywords:** Emotion Dysregulation, Externalizing Disorders, ADHD, Oppositional Defiant Disorder, Conduct Disorder, Children and Adolescents, Meta-Analysis

## Abstract

Externalizing disorders (Attention Deficit Hyperactivity Disorder (ADHD), Oppositional Defiant Disorder (ODD) and Conduct Disorder (CD)) are frequent among children and adolescents referred to mental health services. Despite numerous studies linking emotion dysregulation to both severity and symptoms of externalizing disorders, the strength of the relationship is unclear. The purpose of this systematic review and meta-analysis was to evaluate the correlation between emotion dysregulation (deficits in emotion regulation and emotional lability) and symptoms of externalizing disorders. Embase OVID, Ovid MEDLINE and PsycINFO were systematically searched for eligible studies published up to June 2025. In total 75 studies involving 11,913 participants were included, with 63 studies on ADHD, 9 on ODD, 2 on ODD/CD, and 1 on ADHD/ODD/CD. Results showed a moderate correlation between emotion dysregulation and symptoms of externalizing disorders (*r*=0.35), with a higher correlation for emotional lability (*r*=0.47) than for emotion regulation (*r*=0.24). No significant moderating effects were found for age, gender, continent, informant, ADHD subtype, comorbidity or medication status. The findings indicate that emotion dysregulation, especially emotional lability, has a significant relation to symptoms of externalizing disorders. PROSPERO [CRD420251061853].

**Supplementary Information:**

The online version contains supplementary material available at https://doi.org/10.1007/s00787-026-03090-8.

## Introduction

Symptoms of childhood mental disorders have traditionally been divided into two broader dimensions, externalizing and internalizing, a division which has been widely supported in factor analyses [1, 2]. The externalizing disorders, Attention Deficit Hyperactivity Disorder (ADHD); Oppositional Defiant Disorder (ODD); and Conduct Disorder (CD), are common in clinical samples of children and adolescents and comprise a large proportion of referrals to mental health services. ADHD has a reported prevalence of 7.2%, ODD 3.6% and CD 2.1% and all three disorders are reported to be more prevalent in boys than girls [3, 4]. Comorbidity between these disorders is very high; a recent meta-analysis showed that 34.7% of children and adolescents diagnosed with ADHD are also diagnosed with ODD and 10.7% with CD [5]. Although ADHD is classified as a neurodevelopmental disorder and ODD and CD as disruptive behavior disorders, their classification into externalizing disorders remains relevant as they are all characterized by behavior problems related to impulsivity and rule-breaking behavior. All three disorders are also associated with poorer social functioning and frequent peer problems [6, 7].

Emotion regulation in children and adolescents with externalizing disorders has received increased interest in recent years. Emotion regulation is a complex construct and there is considerable variety in its definition in the literature. The most widely used definition in clinical research on children and adolescents describes emotion regulation as the processes involved in modifying an emotional state, influencing the expression, duration and intensity of emotions [8, 9]. This applies not only to controlling negative emotions, as it encompasses increasing, decreasing or maintaining both negative and positive emotions [8].

Research shows that children acquire basic skills to regulate their emotions between ages 2–5 years, but continue to improve in effortful self-regulation as they mature. In early childhood, support seeking from adults can be the prominent form of regulation, but older children rely on more complex cognitive and behavioral strategies to modulate their emotional reactions [9–12]. One aspect of emotion dysregulation refers to children who have difficulty with managing their emotions, which may change rapidly and/or be expressed intensively. Deficits in emotion regulation are, however, not only related to the acquisition of regulatory strategies as a second aspect of emotion dysregulation is emotional lability, or the child’s sensitivity to eliciting events. Research has shown that children differ in their emotional lability, which refers to the rapidity of responding to emotion eliciting events and how long it takes the child to recover from their reaction [13–16]. This refers to both negative and positive events, as emotional lability can, for example, present both as high excitability to rewards and reactive aggression to provocation [17]. Emotion dysregulation thus refers to a lack of age-appropriate emotion regulation, which can present as emotional lability and/or ineffective emotion regulation [18–20].

Emotion dysregulation has been linked to various types of psychopathology in children and adolescents, both internalizing and externalizing disorders (e.g [21–24]). Within externalizing disorders, many studies have shown a strong link between emotion dysregulation and ODD as well as ADHD, and emotion dysregulation has been reported as a core feature of both disorders [19, 25–29]. In ADHD, emotion dysregulation has been linked to risky behaviors, aggression, substance abuse and social impairment [25, 30]. Studies comparing subtypes of ADHD and emotion dysregulation are lacking, but some evidence suggests that higher levels of emotion dysregulation are found for the combined subtype than the primarily inattentive type [31].

For children with ODD, evidence shows that emotion dysregulation is related to symptom severity, as higher levels of emotion dysregulation predict greater number of symptoms for both the angry/irritable and the argumentative/defiant dimensions [32]. Furthermore, longitudinal studies have shown that the relation between ODD symptoms and emotion dysregulation is stable across time, with emotion regulation negatively correlated with ODD symptoms and emotional lability positively correlated in studies assessing children repeatedly over several years [19, 28]. Research findings also indicate that emotion dysregulation is distinct from ODD symptoms and that the relation is not due to overlap in assessment instruments. In their study, Mitchison et al. [33] conducted an item analysis on the Disruptive Behavior Rating Scale (DBRS), which measures symptoms of ODD, and the Emotion Regulation Checklist (ERC). The study was based on parent report for 609 children and they found overlap (*r* > 0.5) between the DBRS and four out of 15 items on the emotional lability subscale of the ERC. However, results showed that the correlation between the two scales remained significant even after these overlapping items were removed, indicating that the correlation was not attributable to item overlap. Bunford et al. [17] also found that in adolescents with ADHD, emotion dysregulation was distinct from comorbid ODD and was associated with social impairment beyond the effects of ODD. In ADHD and ODD, emotion dysregulation therefore seems to represent a distinct dimension that independently predicts clinical impairment in children and adolescents. For both ADHD and ODD, there also seems to be a stronger relation to emotional lability than deficits in emotion regulation skills. More specifically, many studies have shown a larger effect size or stronger correlation (calculated as Cohen’s *d* or correlation) between symptoms of ADHD or ODD and emotional lability than for emotion regulation (e.g [17, 19, 28, 34]).

Studies targeting emotion regulation in children with CD seem to have been mostly focused on the role of callous-unemotional (CU) traits. However, as CU traits refer to the lack of empathy for other people’s emotions [35] as opposed to the ability to regulate one’s own emotions, it can be argued that CU traits are not a part of emotion dysregulation per se. This distinction between emotion dysregulation and CU traits is supported by studies showing that emotion dysregulation is strongly related to reactive aggression as is often seen in ODD, while CU traits are strongly related to proactive aggression which is more in line with symptoms of CD [36, 37]. However, a few studies have combined children with ODD and CD into one group and reported a higher rate of emotion dysregulation compared to a control group [16, 38, 39], indicating that emotion dysregulation may also be related to symptoms of CD.

Despite a plethora of studies linking emotion dysregulation to externalizing disorders the only previous meta-analysis published on this topic is on the relation between ADHD and emotion dysregulation [34]. In the study by Graziano and Garcia [34], the authors divided emotion dysregulation into four different domains. The first domain, *Emotion Recognition or Understanding* (ERU) referred to the child’s ability to recognize others’ expressed emotions, the second *Emotional Reactivity/Lability/Negativity* (ERNL) to the child’s reactivity (both positive and negative) to an emotion eliciting event, the third *Emotion Regulation* (EREG) to the child’s ability to regulate their emotions, and the fourth *Empathy/Callous-Unemotional Traits* (ECUT) referred to the child’s ability to experience another person’s affective state. The results showed that children and adolescents with ADHD had the most impairment in emotional lability (ERNL) (*d*=0.95) followed by emotion regulation (EREG) (*d*=0.80), but significantly smaller effects were seen for emotion recognition/understanding (ERU) (*d*=0.64) and empathy/callous-unemotional traits (ECUT) (*d*=0.68) [34].

With only one meta-analysis on ADHD and emotion dysregulation, published almost 10 years ago, and none for ODD or CD, a comprehensive systematic review and meta-analysis on emotion dysregulation in externalizing disorders seems warranted. The purpose of this systematic review and meta-analysis was to examine the strength of the relationship between emotion dysregulation and externalizing disorders. To the best of our knowledge, this is the first meta-analysis on externalizing disorders and emotion dysregulation. The definition of emotion dysregulation used for the current study includes emotion regulation and emotional lability, which are congruent with domains 2 and 3 (EREG and ERNL) from Graziano and Garcia [34] meta-analysis on ADHD and emotion dysregulation. Domains 1 and 4 (ERU and ECUT) from that study are left out as they refer to how well the child can recognize and/or experience the emotions of others and not the regulation of emotions. The current meta-analysis thus provides an update on the relation between emotion dysregulation and ADHD from Graziano and Garcia [34] and expands it by the addition of ODD and CD providing valuable information about emotion dysregulation in children and adolescents with externalizing disorders. We also apply a stricter criterion regarding diagnostic procedures by requiring that a diagnostic interview had to be used for a study to be included in the meta-analysis. The current meta-analysis had two specific aims: First, to evaluate the magnitude of the correlation between emotion dysregulation and symptoms of externalizing disorders. Second, to evaluate whether type of disorder, proportion of males, and mean age are associated with different levels of emotion dysregulation. We examined the role of gender since externalizing disorders are more prevalent among boys and because findings suggest that girls have better emotion regulation than boys [3, 4, 40] and we examined the role of age since emotion regulation typically enhances with older age [9].

## Method

### Literature search

The protocol for this meta-analysis was pre-registered at PROSPERO with ID CRD420251061853. The meta-analysis was conducted according to the PRISMA guidelines [41], see online Supplement S1.

We identified relevant studies through a systematic and comprehensive literature search of electronic databases and scanned the reference lists of the included studies. The search strategy was applied to Ovid MEDLINE, Embase OVID, and PsycINFO from the start of the data bases to September 2, 2024. Updated searches were conducted June 19, 2025. The authors, in collaboration with a university librarian who conducted the database searches, generated the list of search items to identify relevant studies. We used both subject headings and free text words for the following search terms to search the databases: (Attention deficit disorders and variations thereof [e.g., ADHD, ADD, Hyperkinetic Disorder], OR Disruptive behavior disorders and variations thereof [e.g., conduct disorder, oppositional defiant disorder, externali* disorder* OR syndrome]) AND (Emotion dysregulation and variations thereof [e.g., emotional lability, frustration intolerance, mood swing, temper outburst, emotional fragility, irritable mood, vindictiveness, temper tantrums, mood dysregulation, emotion regulation OR dysregulation, emotional reactivity OR sensitivity]), AND study design (Epidemiologic OR Cross-sectional OR observational OR Registry OR Registries OR Prevalence OR case-control OR prospective OR retrospective OR case series), AND age group (up to 18 years). The search strategy was peer reviewed by a second university librarian. Duplicate records were removed using a fully automated AI-based deduplication solution, Deduklick [42]. For details on the electronic search see online Supplement 2.

Four pairs of authors (UN & JL, GJW & ENR, KWF & CMP, GJW & AMS) read the abstracts independently of each other to decide whether a study warranted a more detailed reading. Full-text articles were retrieved if there was any indication of a target group of ADHD, CD, or ODD youth patients being assessed for emotion dysregulation. In total, 483 full-text articles were read independently by the authors in each pair and considered for inclusion. The final decision for article inclusion was made using a stricter set of inclusion criteria, and any disagreements were resolved by consensus discussion among the authors or consultation with the last author.

### Inclusion and exclusion criteria

To be included in the meta-analysis an article had to:

1. Be published, or in press, in an English language journal.
2. Have participants diagnosed with ADHD, ODD, or CD according to DSM (III-5) or ICD (10–11) using a diagnostic interview.
3. Use a validated rating scale of emotional dysregulation.
4. Provide a correlation between emotion dysregulation scores and symptoms of externalizing disorders or means (SDs) in a study comparing participants diagnosed with ADHD, ODD, or CD with healthy controls.
5. Have a sample consisting of at least 10 participants.
6. Have participants aged 0–18 years (studies with mean age < 18 years and max age of participants at 20 years may be included).

**Fig. 1:**
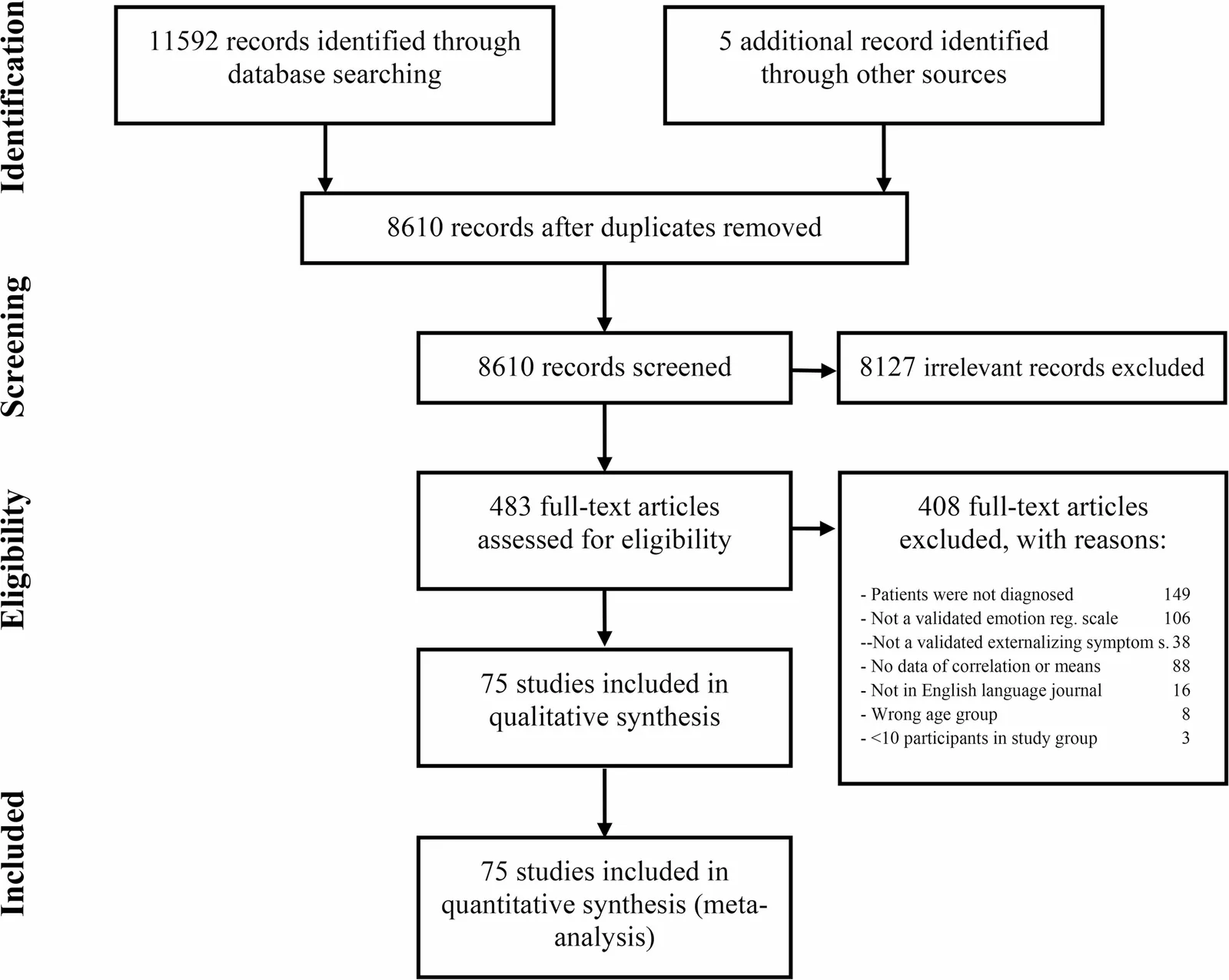
Flow-chart of the inclusion of studies

Studies were excluded if they did not meet one or more of the inclusion criteria, e.g., not diagnosing the participants applying DSM- or ICD-criteria or using an observation measure of emotion dysregulation in a laboratory experiment (see Fig. 1).

### Data extraction

Background data were extracted independently by the two authors in each pair and disagreements were discussed to reach consensus. We extracted information on study variables, such as primary disorder (ADHD, ODD; CD, or combinations thereof), diagnostic interview and system, continent where the study was conducted, type of sample (clinic or community), study design (within or between), emotion dysregulation measure, symptom measure, and informant. We also extracted participant data, e.g., mean age, percent males, subtype of ADHD, percent having a comorbid disorder, percent medicated for the externalizing disorder, race, ethnicity, correlation coefficients, and means and SDs (for comparative studies). A coding scheme and a scoring manual including the variables of interest were developed.

### Potential moderators

Potential moderators to be analyzed were decided a priori. We used five categorical variables that the majority of the studies had information on: *Disorder* (ADHD or CD/ODD), *Design* was either within (one group of children for which emotion dysregulation and externalizing symptoms were assessed and a correlation presented) or between (one group with externalizing disorders was compared with one group of healthy controls on a measure of emotion dysregulation), *Informant* was either parent, parent + child, parent + teacher, or child/adolescent alone, *Emotion dysregulation domain* (EREG or ERNL), and *Continent* (based on the country in which the study was carried out) Africa (A), Asia, Europe (E), Oceania (O), North America (NA), or South America (SA). We also used the following variables as potential continuous moderators: number of participants in the study, mean age of the sample, percent males, and percent diagnosed with the subtypes of ADHD: inattentive, hyperactive-impulsive, and combined, respectively.

### Validated scales measuring emotion dysregulation used in the studies

The most frequently used measure of emotion dysregulation in the included studies was the *Emotion Regulation Checklist* (ERC) [43] which was used in 25 studies (33.3%). Second was the *Emotion Control* subscale of the *Behavior Rating Inventory of Executive Function* (BRIEF) [44] which was used in 14 (18.7%) of the studies; third the *Attention*,* Aggression*,* and Anxious/Depressed Aggregate* from the *Child Behavior Checklist* (CBCL-AAA) [45] used in 11 (14.7%) of the studies; fourth and fifth the *Emotion Lability* subscale of the *Conners’ Parent Rating Scale* (CPRS) [46] and the *Difficulties in Emotion Regulation Scale* (DERS) [47] which were both used in 7 (9.3%) of the included studies. Sixth was the emotion control subscale *Emotion Regulation Index* (ERICA) [48] used in 2 studies (2.7%). Other measures were only used in one of the included studies (1.3% each): *Early Adolescent Temperament Questionnaire—Revised* (EATQ) [49]; *Adolescent Daily Emotion Regulation Questionnaire* (ADERQ) [50]; *Emotional Self-control Scale* from the *Behavior Assessment for Children* (BASC-2) [51]; the *Cyclothymic-Hypersensitive Temperament Questionnaire* (CHTQ) [52]; *Expression and Emotion Scale for Children* (EESC) ([53]; *Emotion Questionnaire* [54]; *Emotion Regulation Questionnaire* [55]; *Reactivity*,* Intensity*,* Polarity and Stability Questionnaire* (RIPoSt-40) [56]; and the *Trait Emotional Intelligence Child* form (TEI) [57].

### Risk of bias

To the best of our knowledge, there is no specific scale to assess risk of bias (RoB) in observational and cross-sectional studies. A measure that approximates the Cochrane RoB assessments for randomized [58] and non-randomized studies [59] is the Quality Assessment Tool for Observational Cohort and Cross-Sectional Studies [60]. It contains 14 items, evaluated as Yes, No, cannot determine (CD), not applicable (NA), or not reported (NR). Since not all items are applicable for all studies, we calculated a percentage of the maximum score possible after removing items evaluated as not applicable. For example, if a study had 9 Yes out of a possible 12, the score would be 75% for that study. Studies having a score of ≤ 50% was classified as poor, 50.1 to 74.9% as fair, and ≥ 75% as good.

### Meta-analysis

The primary measure for this meta-analysis was the correlation (*r*) between a measure of emotion dysregulation and symptoms of an externalizing disorder, reported in studies using a within group design. We also included studies with a between group design, in which participants with a diagnosis of ADHD, CD, or ODD were compared with healthy controls, i.e., not fulfilling criteria for these diagnoses. In the latter studies Cohen’s *d* (standardized mean difference) was calculated and then converted to *r*. All analyses were done in *Comprehensive Meta-analysis*, *version 4* using a random effects model, since it cannot be assumed that the ESs come from the same population. First, the effect sizes (ESs) were transformed to *z*-values using Fisher’s *r* to *z* transformation to do the analyses. Then, the software did a back-transformation to present the pooled ES as *r* values, which makes understanding of the result easier.

Heterogeneity among ESs was assessed with the Q-statistic and the prediction interval (the true effect size in 95% of all comparable populations will fall within this interval). We also included the *I*^*2*^ statistic, i.e., “what proportion of the observed variance reflects variance in true effects rather than sampling error” [61] (p.282).

Pre-planned moderator analyses of continuous variables were done with meta-regression using the random effects model, and of categorical variables with subgroup analysis using the mixed-effect model, both in CMA. For meta-regression analysis we used the criterion of at least 10 studies to include the variable. For the subgroup analyses we only included categories having at least five data points.

Publication bias was analyzed with funnel plot and Egger’s regression intercept. Sensitivity analysis was conducted by leaving one study out at a time and running the analysis without that study.

## Results

The database searches yielded 75 studies of participants (*N* = 11,931) diagnosed with ADHD, CD or ODD, for which the association between emotion dysregulation and symptom level of the externalizing disorder in question could be extracted. References to the included studies can be found in Supplement 3.

### Background variables

Table 1 presents the most important background variables for the included studies. Most studies were carried out in North America (30), followed by Europe (25), Asia (17), Oceania (2), and Africa (1). Regarding disorder ADHD was the most common with 63 studies, whereas ODD had 9, CD/ODD 2, and ADHD/CD/ODD 1. The diagnostic system used in the studies were DSM-III in 1, DSM-IV in 49, DSM-5 in 23, DSM-IV and 5 in 1, and ICD-10 in 1 study.

**Table 1:** Background data for the included studies

Study	Continent	Disorder	Diagnostic system	*N*	Age range	Mean age	% males	ADHD % inatt.	ADHD % H-I		% comorbidity	% medi cated
Aboelella, 2017	Af	ADHD	DSM-IV	50	8–12	9.0	70.0	12.0	26.0	62.0		
Akman, 2024	E	ADHD	DSM-5	100	8–13	10.8	76.0				22.0	
Alacha, 2024	NA	ADHD	DSM-IV	35	7–12	9.8	67.6	17.1	0.0	82.9	22.9	62.9
Al-Yagon, 2017	A	ADHD	DSM-5	50	11–12	11.4	49.0					
Becker, 2020	NA	ADHD	DSM-5	162	12–14	13.2	64.8	74.1	0.0	25.9	45.7	59.3
Breaux, 2021	NA	ADHD	DSM-5	118	15–17	16.0	55.5	72.1	0.0	27.9		
Bunford, 2015	NA	ADHD	DSM-5	171	10–14	12.1	76.0	51.1	0.0	48.9	81.8	
Bunford, 2017	NA	ADHD	DSM-5	48	8–10	9.0	62.5	10.2	0.0	89.8	57.1	
Bunford, 2018	NA	ADHD	DSM-5	284	10–17	14.4	77.1					
Chan 2025	NA	ADHD	DSM-5	134	13–16	14.1	65.0					
Chen, 2022	A	ODD	DSM-IV	275	6–13	9.3	70.9					
Cirey, 2022	E	ADHD	DSM-IV	70	12–17	14.4	72.8					
Cleminshaw, 2020	NA	ADHD	DSM-5	174		14.5	81.4	49.7	0.6	49.7	49.0	43.0
Donfrancesco 2012	E	ADHD	DSM-IV	190	6–17	8.9	87.9	36.3	14.7	49.0		
Dunsmore, 2012	NA	ODD	DSM-IV	72	7–14	9.7	66.6				80.0	
Dunsmore, 2016	NA	ODD	DSM-IV	89	7–15	9.5	60.1				87.3	
Fogleman, 2018	NA	ADHD	DSM-IV	133	7–13	9.0	65.4					39.8
Gair 2022	NA	ADHD	DSM-IV	31	4–7	6.4	74.2					7.4
Gershy 2025	A	ADHD	DSM-5	58	6–14	9.5	72.4	24.1	10.3	65.0		81.0
Graziano, 2011	NA	ADHD	DSM-IV	80	4–18	10.8	77.5	31.3	1.3	63.8		64.0
Graziano, 2013	NA	ADHD	DSM-IV	74	6–17	10.7	78.4	32.4	1.4	63.5		64.0
Hawkey, 2024	NA	ADHD	DSM-IV	72	7–11	8.3	75.0					
He, 2023	A	ODD	DSM-IV	256	6–13	9.6	72.3				32.8	
Hernandez, 2024	NA	ADHD	DSM-IV	323		5.5	68.7					
Hosiri, 2024	A	ADHD	DSM-5	118	6–12	9.5	81.4				51.7	100.0
Hovik, 2017	E	ADHD	DSM-IV	77	8–17	11.6	51.9	57.1	0.0	42.9	24.7	2.6
Huguet, 2019	E	ADHD	DSM-5	72	7–12	8.8	70.6	31.4	4.3	64.3	52.9	0.0
Hulvershorn, 2014	NA	ADHD	DSM-IV	63	6–13	9.4	79.0				27.0	25.0
Izmir, 2024b	E	ADHD	DSM-5	634	8–12	10.0	66.7					
Jaisle, 2023	NA	ADHD	DSM-5	57	8–13	10.5	63.0					29.0
Jarratt 2005	NA	ADHD	DSM-IV	42	9–15	11.8	85.7	33.3	0.0	66.7		50.0
Jensen, 2018	E	ADHD	DSM-IV	41	8–12	9.7	72.5	29.3	7.3	63.4	41.5	0.0
Jiang, 2016	A	ODD	DSM-IV	24	10–14	12.3	95.8				70.8	0.0
Katic, 2013	NA	ADHD	DSM-IV	318	6–12	9.1	70.7					59.3
Kvadsheim, 2020	E	ADHD	DSM-IV	67	11–17	14.5	64.2	38.2	2.9	58.8	29.4	55.9
Leaberry, 2019	NA	ADHD	DSM-IV	40	8–11	9.4	72.5	30.0	2.5	67.5		70.0
Lin, 2018	A	ODD	DSM-IV	248	6–13	9.6	72.1					
Linder 2010	A	ADHD	DSM-IV	80	7–18	12.5	58.7	43.0	5.0	52.0		
Liu 2025	A	ADHD	DSM-IV	148	9–16	11.1	81.7					
Liu, 2020	A	ADHD	DSM-IV	2760	6–16	9.7	68.7	51.2	4.8	44.0	35.6	0.0
Mahone 2007	NA	ADHD	DSM-IV	25	3–6	4.8	80.0					
Maire, 2020	E	ADHD	DSM-IV	108	7–11	8.9	81.0	33.0	0.0	67.0	50.0	14.0
Marques, 2024	E	ADHD	DSM-5	72	6–10	9.0	63.0				32.0	74.0
Masi, 2020	E	ADHD	DSM-5	42	8–16	9.9	88.1					0.0
Masi, 2021	E	ADHD	DSM-5	154	9–18	13.8	67.5				18.8	
Mauri, 2020	E	ADHD	DSM-5	43	6–16	11.0	86.0	31.0	11.0	58.0		0.0
Miranda, 2008	E	ADHD	DSM-IV	72	6–14	9.4	93.1	0.0	0.0	100.0	50.0	48.0
O’Kearney, 2020	O	ODD	DSM-IV	74	4–8	6.0	63.5				55.4	
Ozbaran, 2018	A	ADHD	DSM-IV	100	11–17	14.0	69.5	50.0	0.0	50.0		0.0
Ozyurt, 2017	A	ADHD	DSM-5	61	8–12	9.7	78.7	19.7	31.1	49.2	44.3	0.0
Panteli, 2024	E	External.	DSM-IV	39	10–12	11.2	51.7					0.0
Pate, 2020	O	ODD	DSM-IV	68	4–8	5.9	63.2					
Perwien, 2008	NA	ADHD	DSM-IV	179	6–12	9.4	66.5				40.7	58.7
Pisano, 2022	E	ADHD	DSM-5	70	9–18	13.0	85.7				28.6	
Predescu, 2020	E	ADHD	ICD-10	27	6–11	9.1						
Raschle, 2025	E	ODD, CD	DSM-5	102	13–18	15.1	0.0				28.1	
Ros, 2020	NA	ADHD	DSM-5	32	4–6	4.7	75.0					
Rosen, 2012	NA	ADHD	DSM-IV	27	8–11	9.3	70.4	37.0	0.0	63.0	59.3	51.8
Roy, 2018	NA	ADHD	DSM-IV	18	5–9	7.3	77.8	16.7	11.1	55.6	61.1	
Sanabra, 2022	E	ADHD	DSM-IV/5	108	6–16	9.7	67.2	39.8	5.6	46.3	18.8	43.8
Schoorl, 2016	E	ODD, CD	DSM-IV	65	8–12	10.3	100.0				69.0	37.0
Semrud-C., 2010	NA	ADHD	DSM-IV	49	9–16	10.3	55.1	57.1	0.0	42.9	0.0	0.0
Seymour, 2012	NA	ADHD	DSM-IV	37	10–14	11.8	68.0					68.0
Seymour, 2017	NA	ADHD	DSM-IV	109	8–12	10.1	67.0	22.0	2.7	75.2	38.0	67.0
Sharifi, 2024	A	ADHD	DSM-IV	23	8–15	9.1	26.1					
Sjowall, 2019	E	ADHD	DSM-5	52	4–6	5.9	77.0					40.3
Sobanski, 2010	E	ADHD	DSM-IV	1186	5–18	10.8	87.1	0.0	0.0	100.0	64.0	
Spencer, 2011	NA	ADHD	DSM-III	197	6–18	11.0	55.3					28.9
Sørensen, 2010	E	ADHD	DSM-IV	34	8–11	9.7	82.4				23.5	
Taskiran, 2018	E	ADHD	DSM-IV	48	6–12	8.4	87.5	35.4	8.3	56.6	47.9	0.0
Tsai, 2021a	A	ADHD	DSM-IV	77		11.6	82.0				14.0	13.0
Tsai, 2021b	A	ADHD	DSM-IV	118	7–18	11.1	85.2	50.8	1.7	47.5	18.6	11.9
Uran 2015	E	ADHD	DSM-IV	67	7–18	12.1	69.0	0.0	0.0	100.0		
Wu 2025	A	ADHD	DSM-IV	354	6–15	8.2	85.0	100.0	0.0	0.0		
Zhang, 2023	A	ODD	DSM-IV	256	6–13	9.6	72.7					

Continent: *A* Asia, *Af* Africa, *E* Europe, *NA* North America, *O* Oceania. Disorder: *ADHD* Attention Deficit Hyperactivity Disorder, *CD* Conduct Disorder, *ODD* Oppositional Defiant Disorder. Diagnostic system, *DSM* Diagnostic and statistical manual of mental disorders, *III* third ed., *IV* fourth ed., 5 = fifth ed., *ICD-10* International classification of diseases, 10th ed. Empty cells indicate that the study did not provide information on this variable

The age range in the included studies varied to a large extent. Some studies focused on young children, e.g. Mahone and Hoffman [62] with a range from 3 to 6 years, others had only adolescents, e.g., Raschle et al. [38] with a range of 13–18 years, whereas some included both children and adolescents, e.g., Graziano et al. [63] with a range of 4–18 years. The mean age of the study samples varied from 4.7 to 16.0 years with an overall mean of 10.1 (SD = 2.6). The proportion of males in the samples varied from 0 to 100% with an overall mean of 71.3% (SD = 14.3).

Of the 63 ADHD studies only 54% provided information on the subtypes of this disorder. Across these studies 35.8% had the inattentive type, 4.5% the hyperactive-impulsive type, and 58.8% the combined type. Forty studies (53.3%) reported the proportion of psychiatric comorbidity in their samples and the overall mean was 41.7% (SD = 22.5). However, this is an underestimation since many of the studies did not report “any comorbid disorder” and for these we had to use the disorder with the highest prevalence figure. The proportion of participants receiving drug treatment for their disorder varied from 0 to 100%, with a mean of 35.0% (SD = 29.6). Race-ethnicity data were provided by only 31 studies (41.3%) and these are provided in Supplement 4.

### Risk of bias

The risk of bias evaluation showed that 17 (22.7%) studies had a good quality, 32 (42.7%) had a fair quality, and that 26 (34.7%) studies had a poor quality. The evaluations for the individual studies are presented in Supplement 5.

### Overall effect size

Table 2 presents the overall effect size and separately for the two emotion regulation domains. The overall *r* was 0.35, indicating a significant but moderate correlation between participants’ dysregulation of emotions and severity of their externalizing disorders. This was also the case for the ERNL (0.47) and EREG (0.24) domains and testing the difference between these correlations yielded a non-significant z-value (1.34, *p* = 0.09). All effect sizes had very large and significant heterogeneity. To shed some light on this we conducted a further analysis on the Emotion Regulation Checklist (ERC), which was applied most often in the included studies (k = 25; 33.3%). ERC has one subscale measuring negativity/lability, where a positive correlation with severity is expected, and one measuring emotion regulation skills, where we expect a negative correlation. The subgroup analysis yielded the following results: negativity/lability 0.51 (95% CI 0.42–0.60) and emotion regulation − 0.27 (95% CI -0.41 to -0.13) with a significant difference between them (Q = 73.8, *p* = 0.0001).

**Table 2:** Effect sizes for all studies divided on emotion dysregulation domain

Domain	k	Corr.	95% CI	z-value	95% PI		I^2^
Both domains	89	0.35	0.27–0.43	8.17^b^	-0.42;0.83	2031.9^b^	95.7
ERNL	57	0.47	0.41–0.53	12.79^b^	-0.05;0.79	653.8^b^	91.4
EREG	51	0.24	0.11–0.37	3.40^a^	-0.65;0.85	1296.6^b^	96.1

k = number of studies, *CI* confidence interval, *PI* prediction interval

^a^ = *p* < 0.001, ^b^ = *p* < 0.0001

**Fig. 2:**
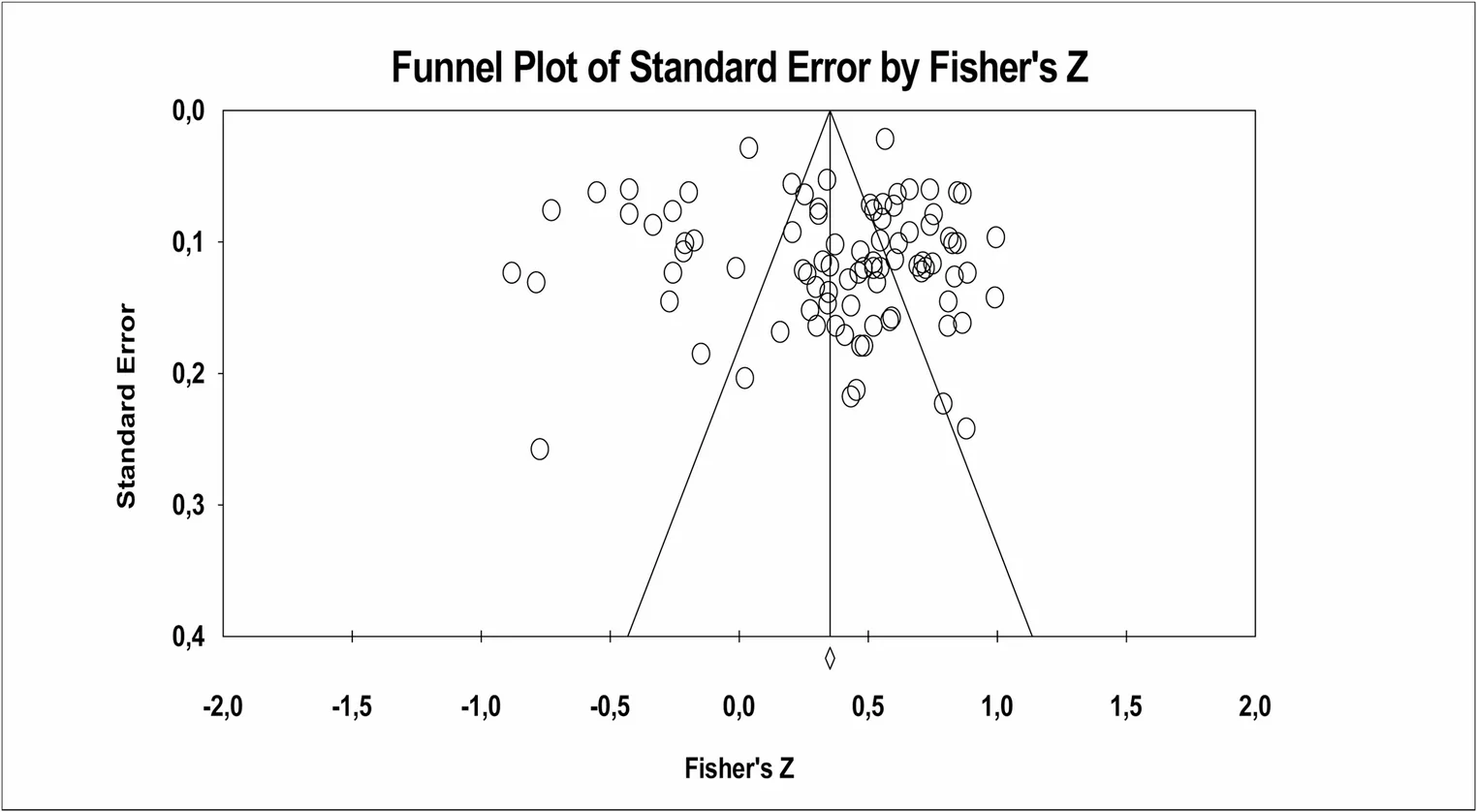
Funnel plot of the included studies

Publication bias regarding the overall ES was analyzed with funnel plot (Fig. 2) and Egger’s regression intercept and yielded a non-significant *t*-value (0.27, *p* = 0.79). Thus, publication bias does not seem to be have affected the meta-analytic results.

Sensitivity analysis was done using the method “one study removed”. This showed that the lowest ES was 0.348 and the highest 0.367. The middle 75% of ESs ranged from 0.350 to 0.360. Thus, the overall ES of this meta-analysis was robust.

### Moderator analyses

The results of the subgroup analyses for the general categorical variables are displayed in Table 3. There was no significant difference between ES with direct *r* and ES with *r* converted from *d*-values. Likewise, the continent in which the study was carried out did not significantly moderate the ES. In this analysis there were too few studies from Oceania (k = 2) and Africa (k = 1) to be included. Finally, whether a parent alone, parent plus child, or parent plus teacher were the informants did not have a significant impact on the ES.

**Table 3:** Subgroup analyses for general categorical variables

Variable	Subgroups	k	Corr.	95% CI	Q-value	*p*-value
*ES data*	Correlations	57	0.31	0.21–0.47	2.06	0.15
	Cohen’s *d* to *r*	31	0.43	0.30–0.54		
*Continent*	North America	40	0.32	0.19–0.44	1.22	0.54
	Europe	29	0.40	0.26–0.53		
	Asia	17	0.42	0.23–0.57		
*Informant*	Parent	43	0.38	0.27–0.49	1.64	0.44
	Parent + child	26	0.28	0.12–0.42		
	Parent + teacher	16	0.41	0.22–0.57		

k = number of studies, *CI* confidence interval

Table 4 contains the results of subgroup analyses testing the effect of diagnosis and whether or not possibly confounding variables were controlled for in the studies’ statistical analyses. Regarding diagnosis, ADHD was associated with a somewhat higher ES than CD/ODD on both domains combined and emotion regulation, but on negativity/lability the disorders had almost exactly the same ES. Concerning control of confounding variables, it would be assumed that studies using such control would have lower ES. However, this was not the case, and on both domains combined and the individual domains studies applying control had nominally, but not significantly, higher ES.

**Table 4:** Subgroup analyses of the effect of diagnosis (upper part) and control of confounding variables (lower part) on the ES for emotion dysregulation domains

Variable	Subgroups	k	Corr.	95% CI	Q-value	*p*-value
Both domains	ADHD	73	0.38	0.30–0.46	2.44	0.11
	CD/ODD	16	0.22	0.01–0.40		
ERNL	ADHD	49	0.47	0.40–0.53	0.004	0.95
	CD/ODD	8	0.48	0.31–0.62		
EREG	ADHD	40	0.29	0.13–0.43	1.48	0.23
	CD/ODD	11	0.08	-0.22;0.37		
*Control of confounders*						
Both domains	Yes	49	0.40	0.29–0.49	1.51	0.22
	No	40	0.30	0.18–0.41		
ERNL	Yes	31	0.49	0.41–0.56	0.34	0.56
	No	26	0.45	0.36–0.54		
EREG	Yes	31	0.32	0.16–0.47	2.33	0.13
	No	20	0.11	0.11–0.32		

k = number of studies, *CI* confidence interval

### Meta-regression analyses

Table 5 presents the results of the meta-regression analyses of the continuous variables. None of the general variables or the specific ADHD variables showed a significant association with the ES.

**Table 5:** Meta-regression analyses of continuous variables

Variable	k	Coefficient	z-value	*p*-value
Number of participants	89	-0.0000	-0.15	0.88
Mean age of sample	88	-0.0186	-0.91	0.36
Percent males	87	0.0030	0.86	0.40
ADHD % inattentive	39	0.0024	0.75	0.45
ADHD % hyperactive-impulsive	39	-0.0113	-1.26	0.21
ADHD % combined	39	-0.0008	-0.27	0.79
Percent comorbidity	40	-0.0077	-0.91	0.36
Percent medicated	26	0.0026	0.52	0.60

k = number of studies

## Discussion

### General results

The primary aim of this meta-analysis was to evaluate the strength of the relation between symptoms of externalizing disorders and emotion dysregulation. Our main findings show that the overall correlation is moderate, with a stronger relation to emotional lability than to emotion regulation. This is consistent with previous findings for both symptoms of ADHD and symptoms of ODD [19, 28, 34]. In the only previously conducted meta-analysis on emotion dysregulation and symptoms of ADHD [34], findings indicated large effect sizes for both emotion lability and emotion regulation. To compare our results directly to these previous findings we converted the present results to effect sizes and found similar results for emotional lability (*d* = 1.07 compared to Graziano & Garcia’s *d* = 0.95) but a slightly lower effect size for emotion regulation (*d* = 0.50 compared to Graziano & Garcia’s *d* = 0.80). This may be due to the current meta-analysis including studies on ADHD, ODD and CD whereas Graziano and Garcia [34] only included studies on ADHD. The current study also had a stricter inclusion criterion by requiring a diagnostic interview for all diagnoses. The present results did show a somewhat larger effect size for ADHD than ODD/CD for overall emotion dysregulation as well as for emotion regulation, but for emotional lability the results showed an equally large effect size for both ADHD and ODD/CD. This is congruent with the results from Bunford et al. [17] who found that emotion dysregulation in adolescents with ADHD was not explained by comorbid ODD.

The results indicate that emotional lability has a moderate relation to symptoms of externalizing disorders. Emotional lability refers to the child reacting swiftly to emotion eliciting events and taking a longer time to recover than their peers. This emotional excitability can apply to both negative and positive reactions and can contribute to difficulties in social interactions [17]. With a weaker relation found for emotion regulation, it seems that emotion dysregulation in externalizing disorders is less related to the lack of age-appropriate emotion regulation skills than the rapidity and duration of the child’s emotional reactions. This infers that reactive aggression and excessive positive excitability may be the core of emotion dysregulation in externalizing disorders.

### Moderator analyses

We did not find any significant moderators as results did not differ based on type of study, informant, or continent. We also did not find any effects of mean age, gender, comorbidity, ADHD subtype or medication status. This indicates that the relation between emotion dysregulation and externalizing disorders is stable and not significantly influenced by other variables.

The fact that gender was not a significant moderator is consistent with previous findings, suggesting that gender does not affect the relation between emotion dysregulation and symptoms of both ADHD and ODD (e.g [33, 34]). Previous results regarding ADHD subtype have been inconsistent, with some studies finding that the relation to emotion dysregulation is stronger in children with the combined subtype [31], whereas others have found no effect of subtype [17]. However, the findings that the relation was not affected by age was somewhat unexpected as the results from a previous meta-analysis on children and adolescents with ADHD indicated a stronger relation between emotional lability and symptoms in samples of older children [34]. This may potentially be explained by the fact that the present meta-analysis had stricter inclusion criteria regarding diagnoses and assessment of emotion dysregulation. However, the samples studied were otherwise quite comparable as the majority of the included studies in the present meta-analysis assessed samples of children with ADHD and the age range was quite large. Finally, medication status (% on medication) was not found to have a significant moderating effect on the relation between symptoms of externalizing disorders and emotion dysregulation. However, as only 26 of the included studies (34%) provided information on medication status, the present results should be interpreted with caution. While several studies have documented the effects of pharmacological interventions on emotion dysregulation in children and adolescents with ADHD, both with and without comorbid ODD and CD [64–66], studies on whether medication status moderates the association between ADHD and emotion dysregulation are lacking and more research is needed to clarify this issue.

### Strengths and limitations

The present systematic review and meta-analysis has several strengths. First it includes 75 studies and a total of 11,931 children and adolescents diagnosed with ADHD, ODD and/or CD and is thus robust. Second, inclusion criteria required a diagnostic interview for both ADHD/ODD/CD and any comorbid diagnoses. Third, screening of all studies and extraction of all information from the included studies was conducted independently and blinded in pairs of co-authors and any discrepancies solved in consensus meetings. Fourth, the rating of risk of bias was conducted by two authors, first by one author and then a random sample independently and blinded by another. Fifth, few significant moderators were found, which is encouraging from a methodological aspect, as confidence in the overall results increases when child gender, age, and ADHD subtype do not seem to affect the correlation between emotion dysregulation and externalizing disorder. Sixth, assessment of publication bias regarding the overall ES was not significant, which is also methodologically encouraging.

This systematic review and meta-analysis has also some limitations that need to be considered when interpreting the results. First, the quality of a meta-analysis is always restricted by the quality of the original studies and is therefore limited by the data it summarizes. The risk of bias evaluation showed that only 22.7% of studies included in this meta-analysis were classified as having good quality. Second, the externalizing disorders were not represented equally as most studies reported data on ADHD and very few studies reported data regarding symptoms of CD. Third, data on comorbidity, as well as ADHD subtypes and medication use was lacking in many of the included studies which limits the generalizability of findings regarding these variables. Fourth, only studies published in English were included and, consequently, any relevant studies reported in other languages were missed.

### Conclusion and future directions

The findings from this meta-analysis demonstrate a moderate relation between emotion dysregulation and symptoms of externalizing disorders. Emotional lability had a stronger relation to symptoms than emotion regulation both for ADHD and ODD/CD. The relation was stronger for overall emotion dysregulation and emotion regulation in children and adolescents with ADHD compared to ODD/CDD, but for emotional lability the effect sizes were equally strong. No significant moderators were found, indicating that the relation is not affected by demographic variables, informant, continent, ADHD subtype or medication status. Future studies should aim to provide information regarding ADHD subtype as this information was often lacking. The results indicate that emotion dysregulation, particularly emotional lability, is a significant factor in all three externalizing disorders and should be considered as part of assessment at the time of diagnosis. As studies have shown that emotion dysregulation contributes to impairment beyond the disorders’ symptoms, studies should also assess whether emotion dysregulation should be given increased attention in the development of treatment for children and adolescents with externalizing disorders.

## Supplementary Information

Below is the link to the electronic supplementary material.

Supplementary Material 1

## Data Availability

No datasets were generated or analysed during the current study.
