# Supplementary material for: Emotion dysregulation in children and adolescents with externalizing disorders: a systematic review and meta-analysis
Source: Eur Child Adolesc Psychiatry. 2026 Jun 16;35(9):2745–57. doi: 10.1007/s00787-026-03090-8 (PMC13695614; doi:10.1007/s00787-026-03090-8)
Supplement: Supplementary file 1 [file 787_2026_3090_MOESM1_ESM.pdf]

Supplement to Njardvik, Wergeland, Fjermestad, Liber, Prentice, Riise, Syversen & Öst

S1. PRISMA Checklist
S2. Literature Search Strategies
S3. References to Included Studies
S4. Race-ethnicity in the included studies
S5. Risk of Bias Evaluation

S1. PRISMA Checklist

Section/topic	#	Checklist item	Reported on page #
**TITLE**			
Title	1	Identify the report as a systematic review, meta-analysis, or both.	1
**ABSTRACT**			
Structured summary	2	Provide a structured summary including, as applicable: background; objectives; data sources; study eligibility criteria, participants, and interventions; study appraisal and synthesis methods; results; limitations; conclusions and implications of key findings; systematic review registration number.	2
**INTRODUCTION**			
Rationale	3	Describe the rationale for the review in the context of what is already known.	3-7
Objectives	4	Provide an explicit statement of questions being addressed with reference to participants, interventions, comparisons, outcomes, and study design (PICOS).	7
**METHODS**			
Protocol and registration	5	Indicate if a review protocol exists, if and where it can be accessed (e.g., Web address), and, if available, provide registration information including registration number.	2, 8
Eligibility criteria	6	Specify study characteristics (e.g., PICOS, length of follow-up) and report characteristics (e.g., years considered, language, publication status) used as criteria for eligibility, giving rationale.	8
Information sources	7	Describe all information sources (e.g., databases with dates of coverage, contact with study authors to identify additional studies) in the search and date last searched.	8
Search	8	Present full electronic search strategy for at least one database, including any limits used, such that it could be repeated.	Supplement S2

Section/topic	#	Checklist item	Reported on page #
Study selection	9	State the process for selecting studies (i.e., screening, eligibility, included in systematic review, and, if applicable, included in the meta-analysis).	8-9, Figure 1, Supplement S3
Data collection process	10	Describe method of data extraction from reports (e.g., piloted forms, independently, in duplicate) and any processes for obtaining and confirming data from investigators.	9-10
Data items	11	List and define all variables for which data were sought (e.g., PICOS, funding sources) and any assumptions and simplifications made.	9-10
Risk of bias in individual studies	12	Describe methods used for assessing risk of bias of individual studies (including specification of whether this was done at the study or outcome level), and how this information is to be used in any data synthesis.	11
Summary measures	13	State the principal summary measures (e.g., risk ratio, difference in means).	11-12
Synthesis of results	14	Describe the methods of handling data and combining results of studies, if done, including measures of consistency (e.g., I^2) for each meta-analysis.	12
Risk of bias across studies	15	Specify any assessment of risk of bias that may affect the cumulative evidence (e.g., publication bias, selective reporting within studies).	11-12
Additional analyses	16	Describe methods of additional analyses (e.g., sensitivity or subgroup analyses, meta-regression), if done, indicating which were pre-specified.	12
**RESULTS**			
Study selection	17	Give numbers of studies screened, assessed for eligibility, and included in the review, with reasons for exclusions at each stage, ideally with a flow diagram.	13, Figure 1, Supplement S3
Study characteristics	18	For each study, present characteristics for which data were extracted (e.g., study size, PICOS, follow-up period) and provide the citations.	13, Table 1
Risk of bias within studies	19	Present data on risk of bias of each study and, if available, any outcome-level assessment (see Item 12).	14, Supplement S5

Section/topic	#	Checklist item	Reported on page #
Results of individual studies	20	For all outcomes considered (benefits or harms), present, for each study: (a) simple summary data for each intervention group and (b) effect estimates and confidence intervals, ideally with a forest plot.	13-15, Table 2-4, Supplement S4-S5
Synthesis of results	21	Present results of each meta-analysis done, including confidence intervals and measures of consistency.	13-15, Table 2-4, Supplement S4-S5
Risk of bias across studies	22	Present results of any assessment of risk of bias across studies (see Item 15).	Supplement S5
Additional analysis	23	Give results of additional analyses, if done (e.g., sensitivity or subgroup analyses, meta-regression [see Item 16]).	15, Table 3-4
**DISCUSSION**			
Summary of evidence	24	Summarize the main findings including the strength of evidence for each main outcome; consider their relevance to key groups (e.g., health care providers, users, and policy makers).	16-19
Limitations	25	Discuss limitations at study and outcome level (e.g., risk of bias), and at review level (e.g., incomplete retrieval of identified research, reporting bias).	18
Conclusions	26	Provide a general interpretation of the results in the context of other evidence, and implications for future research.	19
**FUNDING**			
Funding	27	Describe sources of funding for the systematic review and other support (e.g., supply of data); role of funders for the systematic review.	Enclosed document

S2. Literature Search Strategies

Database: Ovid MEDLINE(R) and Epub Ahead of Print, In-Process, In-Data-Review & Other Non-Indexed Citations, Daily and Versions <1946 to June 18, 2025>; Search date 19 June 2025

1 exp "Attention Deficit and Disruptive Behavior Disorders"/ 42448
2 (attention deficit* adj2 disorder*).ti,kf. 19221
3 ((hyperkinetic or oppositional defiant or disruptive behavio* or impulsiv or inattent* or externali?ing) adj1 (disorder* or syndrome)).ti,kf. 1715
4 (Minimal Brain Dysfunction* or Minimal Brain Disorder* or Conduct Disorder* or Callous-Unemotional Trait*).ti,kf. 2796
5 (ADHD or ADDH or ADHS or ad-hd or hkd).ti,kf. 19040
6 1 or 2 or 3 or 4 or 5 50137
7 Emotional Regulation/ 3869
8 Emotional Adjustment/ 1103
9 Emotional intelligence/ or empathy/ 27988
10 Irritable Mood/ 2193
11 Frustration/ 2493
12 anger/ 9392
13 ((Emotion* or affect* or mood or mental or psychic* or psychological) adj1 (dysregulation or regulation or "self regulation*" or self-regulation or control or managing or modulating or lability or instability or stability or swing)).ti,ab,kf. 36758
14 ((Emotion* adj1 (fragility or awareness or sensitivity or reactivity or adjustment or maladjustment or response* or competen* or knowledge or intelligence or coaching or support)) or empath*).ti,ab,kf. 56561
15 (anger control or anger management or frustration intolerance or temper outburst* or vindictiveness or negative affect or irritability or anger or irritable mood or angry mood or tantrum*).ti,ab,kf. 47448
16 ((argumentative or defiant or oppositional or antagonistic) adj1 behavio?r).ti,ab,kf. 717
17 7 or 8 or 9 or 10 or 11 or 12 or 13 or 14 or 15 or 16 154068
18 6 and 17 3170
19 limit 18 to "all child (0 to 18 years)" 2281
20 limit 18 to "all adult (19 plus years)" 764
21 18 not (20 not 19) 2894
22 limit 21 to (meta analysis or "review" or "systematic review") 410
23 21 not 22 2484

https://ovidsp.ovid.com/ovidweb.cgi?T=JS&NEWS=N&PAGE=main&SHAREDSEARCHID=1DAq7tgpgNSo9Jrn01RvqvE4XKauqEkIZ5My9NORAMpLIxKSR8qX4LkD6Xl0oZd1b

Comments:
/ indicates that the search term is a subject heading
.ti,ab,kf. Search term in title, abstract, or author keywords
Adj proximity operator indicates that search terms are close to each other. Adj2 means that search term two is the first or second (2) word after search term one, or vice versa
Line 21: removed articles indexed with adults (20), but not indexed with children (19)

Database: Embase (Ovid) <1974 to 2025 June 18>; Search date 20 June 2025

1 attention deficit disorder/ or exp disruptive behavior/ or externalizing disorder/ or conduct disorder/ 112130
2 (attention deficit* adj2 disorder*).ti,kf. 26619
3 ((hyperkinetic or oppositional defiant or disruptive behavio* or impulsiv or inattent* or externali?ing) adj1 (disorder* or syndrome)).ti,kf. 2565
4 (Minimal Brain Dysfunction* or Minimal Brain Disorder* or Conduct Disorder* or Callous-Unemotional Trait*).ti,kf. 3821
5 (ADHD or ADDH or ADHS or ad-hd or hkd).ti,kf. 29888
6 1 or 2 or 3 or 4 or 5 115766
7 emotion regulation/ 11797
8 mental instability/ 7459
9 emotional stability/ 8530
10 Emotional intelligence/ 5044
11 emotional awareness/ 356
12 empathy/ 37659
13 emotional support/ 4447
14 Irritability/ 34548
15 frustration/ 12066
16 anger/ 25306
17 ((Emotion* or affect* or mood or mental or psychic* or psychological) adj1 (dysregulation or regulation or "self regulation*" or self-regulation or control or managing or modulating or lability or instability or stability or swing)).ti,ab,kf. 49587
18 ((Emotion* adj1 (fragility or awareness or sensitivity or reactivity or adjustment or maladjustment or response* or competen* or knowledge or intelligence or coaching or support)) or empath*).ti,ab,kf. 70225
19 (anger control or anger management or frustration intolerance or temper outburst* or vindictiveness or negative affect or irritability or anger or irritable mood or angry mood or tantrum*).ti,ab,kf. 65804
20 ((argumentative or defiant or oppositional or antagonistic) adj1 behavio?r).ti,ab,kf. 923
21 7 or 8 or 9 or 10 or 11 or 12 or 13 or 14 or 15 or 16 or 17 or 18 or 19 or 20 239254
22 6 and 21 10473
23 limit 22 to child <unspecified age> 5317
24 limit 22 to (adult <18 to 64 years> or aged <65+ years>) 3401
25 22 not (24 not 23) 8369
26 limit 25 to (chapter or conference abstract) 1602
27 25 not 26 6767
28 limit 27 to ((meta analysis or "systematic review") and "review") 266
29 27 not 28 6501
30 limit 29 to "clinical trial (clinicaltrials.gov)" 382
31 29 not 30 6119

https://ovidsp.ovid.com/ovidweb.cgi?T=JS&NEWS=N&PAGE=main&SHAREDSEARCHID=6YSGgWRaxWHR9r9JZyLOldUAl8yF7Qbut2Ddhbl2sKuxRm9ZPlf6hPwIFKbv927HA

Comments: New from May this year is that EMBASE also includes records from clinicaltrials.gov (see: Elsevier adds 500K clinical trial records to Embase to enable comprehensive search for R&D).

Database: APA PsycInfo (OVID) <1806 to June 2025 Week 2>; Search date 19 June 2025

1 exp attention deficit disorder/ or exp disruptive behavior disorders/ 40109
2 (attention deficit* adj2 disorder*).tw. 37388
3 ((hyperkinetic or oppositional defiant or disruptive behavio* or impulsiv or inattent* or externali?ing) adj1 (disorder* or syndrome)).tw. 7463
4 (Minimal Brain Dysfunction* or Minimal Brain Disorder* or Conduct Disorder* or Callous-Unemotional Trait*).tw. 10276
5 (ADHD or ADDH or ADHS or ad-hd or hkd).tw. 37258
6 1 or 2 or 3 or 4 or 5 57342
7 emotional regulation/ 19142
8 emotional control/ or anger control/ 5451
9 emotional instability/ 607
10 emotional stability/ 1523
11 emotional adjustment/ 17704
12 emotional responses/ 20253
13 emotional intelligence/ 7792
14 empathy/ 18949
15 irritability/ 1502
16 frustration/ 3329
17 tantrums/ 236
18 ((Emotion* or affect* or mood or mental or psychic* or psychological) adj1 (dysregulation or regulation or "self regulation*" or self-regulation or control or managing or modulating or lability or instability or stability or swing)).tw. 48302
19 ((Emotion* adj1 (fragility or awareness or sensitivity or reactivity or adjustment or maladjustment or response* or competen* or knowledge or intelligence or coaching or support)) or empath*).tw. 86270
20 (anger control or anger management or frustration intolerance or temper outburst* or vindictiveness or negative affect or irritability or anger or irritable mood or angry mood or tantrum*).tw. 66408
21 ((argumentative or defiant or oppositional or antagonistic) adj1 behavio?r).tw. 982
22 7 or 8 or 9 or 10 or 11 or 12 or 13 or 14 or 15 or 16 or 17 or 18 or 19 or 20 or 21 220174
23 6 and 22 4729
24 limit 23 to (100 childhood <birth to age 12 yrs> or 200 adolescence <age 13 to 17 yrs>) 3073
25 limit 23 to "300 adulthood <age 18 yrs and older>" 1670
26 23 not (25 not 24) 4007
27 limit 26 to ("0200 book" or "0240 authored book" or "0280 edited book" or "0300 encyclopedia") 587
28 26 not 27 3420
29 limit 28 to (dissertation or reviews) 431
30 28 not 29 2989

https://ovidsp.ovid.com/ovidweb.cgi?T=JS&NEWS=N&PAGE=main&SHAREDSEARCHID=6tAn5cAxKnTycVxSIpQhYyQ9TctuXYbp7UQvKF4WLTYATA3bdekrmpG5ZyxbzxpUT

S3. References to included studies

Abo Elella, E., Hassan, G. A., Sabry, W., Hendawy, H., Shorub, E., Zyada, F., & Medany, O. (2017). Trait emotional intelligence in a sample of Egyptian children with attention deficit hyperactivity disorder. *Child and Adolescent Mental Health*, *22*(4), 216-223.

Akman, H., Serdengeçti, N., Yavuz, M., Kadak, M. T., Ercan, O., & Doğangün, B. (2024). Attachment and comorbid anxiety in ADHD. *Clinical Child Psychology and Psychiatry*, *29*(1), 368-380.

Alacha, H. F., Rosen, P. J., & Bufferd, S. J. (2024). Positive Affect Variability is Associated with Homework Management Difficulties in Children with ADHD. *Journal of Child and Family Studies*, *33*(9), 2933-2946.

Al-Yagon, M., Forte, D., & Avrahami, L. (2020). Executive functions and attachment relationships in children with ADHD: Links to externalizing/internalizing problems, social skills, and negative mood regulation. *Journal of attention disorders*, *24*(13), 1876-1890.

Becker, S. P., Tamm, L., Epstein, J. N., & Beebe, D. W. (2020). Impact of sleep restriction on affective functioning in adolescents with attention-deficit/hyperactivity disorder. *Journal of Child Psychology and Psychiatry*, *61*(10), 1160-1168.

Breaux, R., Dvorsky, M. R., Marsh, N. P., Green, C. D., Cash, A. R., Shroff, D. M., ... & Becker, S. P. (2021). Prospective impact of COVID-19 on mental health functioning in adolescents with and without ADHD: Protective role of emotion regulation abilities. *Journal of Child Psychology and Psychiatry*, *62*(9), 1132-1139

Bunford, N., Evans, S. W., Becker, S. P., & Langberg, J. M. (2015). Attention-deficit/hyperactivity disorder and social skills in youth: A moderated mediation model of emotion dysregulation and depression. *Journal of abnormal child psychology*, *43*(2), 283-296.

Bunford, N., Evans, S. W., Zoccola, P. M., Owens, J. S., Flory, K., & Spiel, C. F. (2017). Correspondence between heart rate variability and emotion dysregulation in children, including children with ADHD. *Journal of abnormal child psychology*, *45*(7), 1325-1337.

Bunford, N., Dawson, A. E., Evans, S. W., Ray, A. R., Langberg, J. M., Owens, J. S., ... & Allan, D. M. (2020). The difficulties in emotion regulation scale–parent report: A psychometric investigation examining adolescents with and without ADHD. *Assessment*, *27*(5), 921-940.

Chan, E. S., Green, C. D., Dvorsky, M. R., Steinberg, A. H., Becker, S. P., & Langberg, J. M. (2025). What dimensions of school climate promote adaptive functioning in adolescents with ADHD? A prospective longitudinal study. *Research in developmental disabilities*, *157*, 104903.

Chen, H., He, T., Xu, M., Zhao, J., Li, L., & Lin, X. (2022). Children's oppositional defiant disorder symptoms make parents difficult to be nice: Longitudinal association among parent emotion regulation, child emotion regulation and children's oppositional defiant disorder symptoms in Chinese children with oppositional defiant disorder. *Clinical Child Psychology and Psychiatry*, *27*(4), 1155-1169.

Cleminshaw, C. L., DuPaul, G. J., Kipperman, K. L., Evans, S. W., & Owens, J. S. (2020). Social deficits in high school students with attention-deficit/hyperactivity disorder and the role of emotion dysregulation. *School psychology*, *35*(4), 233.

Donfrancesco, R., Innocenzi, M., Marano, A., & Biederman, J. (2015). Deficient emotional self-regulation in ADHD assessed using a unique profile of the Child Behavior Checklist (CBCL) replication in an Italian study. *Journal of Attention Disorders*, *19*(10), 895-900

Dunsmore, J. C., Booker, J. A., & Ollendick, T. H. (2013). Parental emotion coaching and child emotion regulation as protective factors for children with oppositional defiant disorder. *Social Development*, *22*(3), 444-466.

Dunsmore, J. C., Booker, J. A., Ollendick, T. H., & Greene, R. W. (2016). Emotion socialization in the context of risk and psychopathology: Maternal emotion coaching predicts better treatment outcomes for emotionally labile children with oppositional defiant disorder. *Social Development*, *25*(1), 8-26

Fogleman, N. D., Slaughter, K. E., Rosen, P. J., Leaberry, K. D., & Walerius, D. M. (2019). Emotion regulation accounts for the relation between ADHD and peer victimization. *Journal of Child and Family Studies*, *28*(9), 2429-2442.

Gair, S. L., Brown, H. R., Breaux, R., Lugo-Candelas, C. I., McDermott, J. M., & Harvey, E. A. (2022). Neural activity and emotion socialization as predictors of later emotion regulation difficulties in children with and without hyperactivity/impulsivity. *Journal of Attention Disorders*, *26*(12), 1668-1681.

Gershy, N., Schorr-Sapir, I., Krinsky, N., & Ne'eman-Ishay, H. (2025). The protective role of parental interpersonal emotion regulation among Israeli families of children with attention-deficit/hyperactivity disorder. *Journal of Family Psychology*.

Graziano, P. A., McNamara, J. P., Geffken, G. R., & Reid, A. (2011). Severity of children's ADHD symptoms and parenting stress: A multiple mediation model of self-regulation. *Journal of abnormal child psychology*, *39*(7), 1073-1083.

Graziano, P. A., McNamara, J. P., Geffken, G. R., & Reid, A. M. (2013). Differentiating co-occurring behavior problems in children with ADHD: patterns of emotional reactivity and executive functioning. *Journal of Attention Disorders*, *17*(3), 249-260.

Hawkey, E. J., Williams, A. I., Chung, S., Owens, E. B., & Pfiffner, L. J. (2024). Emotion Regulation and Organizational Skills in Children With ADHD Symptoms Are Associated With Behavioral Parent Training Adherence. *Journal of Attention Disorders*, *28*(9), 1320-1330.He, 2023

Hernandez, M. L., Garcia, A. M., Spiegel, J. A., Dick, A. S., & Graziano, P. A. (2024). Multimodal assessment of emotion dysregulation in children with and without ADHD and Disruptive behavior disorders. *Journal of Clinical Child & Adolescent Psychology*, *53*(3), 444-459.Hosiri, 2024

Hovik, K. T., Egeland, J., Isquith, P. K., Gioia, G., Skogli, E. W., Andersen, P. N., & Øie, M. (2017). Distinct patterns of everyday executive function problems distinguish children with Tourette syndrome from children with ADHD or autism spectrum disorders. *Journal of attention disorders*, *21*(10), 811-823.

Huguet, A., Eguren, J. I., Miguel-Ruiz, D., Vallés, X. V., & Alda, J. A. (2019). Deficient emotional self-regulation in children with attention deficit hyperactivity disorder: Mindfulness as a useful treatment modality. *Journal of Developmental & Behavioral Pediatrics*, *40*(6), 425-431.

Hulvershorn, L. A., Mennes, M., Castellanos, F. X., Di Martino, A., Milham, M. P., Hummer, T. A., & Roy, A. K. (2014). Abnormal amygdala functional connectivity associated with emotional lability in children with attention-deficit/hyperactivity disorder. *Journal of the American Academy of Child & Adolescent Psychiatry*, *53*(3), 351-361.

Inci Izmir, S. B., Aktan, Z. D., & Ercan, E. S. (2024). The Comparison of Psychological Factors and Executive Functions of Children with Attention Deficit Hyperactivity Disorder and Cognitive Disengagement Syndrome to ADHD and ADHD Comorbid with Oppositional Defiant Disorder. *Journal of Attention Disorders*, *28*(12), 1555-1576.

Jaisle, E. M., Groves, N. B., Black, K. E., & Kofler, M. J. (2023). *Linking ADHD and ASD symptomatology with social impairment: The role of emotion dysregulation. Research on Child and Adolescent Psychopathology, 51 (1), 3–16*.

Jarratt, K. P., Riccio, C. A., & Siekierski, B. M. (2005). Assessment of attention deficit hyperactivity disorder (ADHD) using the BASC and BRIEF. *Applied Neuropsychology*, *12*(2), 83-93.

Jensen, D. A., Høvik, M. F., Monsen, N. J. N., Eggen, T. H., Eichele, H., Adolfsdottir, S., ... & Sørensen, L. (2018). Keeping emotions in mind: The influence of working memory capacity on parent-reported symptoms of emotional lability in a sample of children with and without ADHD. *Frontiers in psychology*, *9*, 1846.

Jiang, W., Li, Y., Du, Y., & Fan, J. (2016). Emotional regulation and executive function deficits in unmedicated Chinese children with oppositional defiant disorder. *Psychiatry investigation*, *13*(3), 277.

Katic, A., Dirks, B., Babcock, T., Scheckner, B., Adeyi, B., Richards, C., & Findling, R. L. (2013). Treatment outcomes with lisdexamfetamine dimesylate in children who have attention-deficit/hyperactivity disorder with emotional control impairments. *Journal of child and adolescent psychopharmacology*, *23*(6), 386-393.

Kvadsheim, E., Fasmer, O. B., Osnes, B., Koenig, J., Adolfsdottir, S., Eichele, H., ... & Sørensen, L. (2020). Lower cardiac vagal activity predicts self-reported difficulties with emotion regulation in adolescents with ADHD. *Frontiers in psychiatry*, *11*, 244.

Leaberry, K. D., Rosen, P. J., Slaughter, K. E., Reese, J., & Fogleman, N. D. (2019). Temperamental negative affect, emotion-specific regulation, and concurrent internalizing and externalizing pathology among children with ADHD. *ADHD Attention Deficit and Hyperactivity Disorders*, *11*(3), 311-324.

Lin, X., Li, L., Heath, M. A., Chi, P., Xu, S., & Fang, X. (2018). Multiple levels of family factors and oppositional defiant disorder symptoms among Chinese children. *Family process*, *57*(1), 195-210.

Linder, N., Kroyzer, N., Maeir, A., Wertman-Elad, R., & Pollak, Y. (2010). Do ADHD and executive dysfunctions, measured by the Hebrew version of Behavioral Rating Inventory of Executive Functions (BRIEF), completely overlap?. *Child Neuropsychology*, *16*(5), 494-502.

Liu, L., Chen, W., Sun, L., Cheng, J., Su, Y., Rudaizky, D., ... & Wang, Y. F. (2020). The characteristics and age effects of emotional lability in ADHD children with and without oppositional defiant disorder. *Journal of Attention Disorders*, *24*(14), 2042-2053.

Mahone, E. M., & Hoffman, J. (2007). Behavior ratings of executive function among preschoolers with ADHD. *The Clinical Neuropsychologist*, *21*(4), 569-586.

Maire, J., Galera, C., Bioulac, S., Bouvard, M., & Michel, G. (2020). Emotional lability and irritability have specific associations with symptomatology in children with attention deficit hyperactivity disorder. *Psychiatry research*, *285*, 112789.

Marques, S., Correia-de-Sá, T., Guardiano, M., Sampaio-Maia, B., & Ferreira-Gomes, J. (2024). Emotion dysregulation and depressive symptoms mediate the association between inhibitory control difficulties and aggressive behaviour in children with ADHD. *Frontiers in psychiatry*, *15*, 1329401.

Masi, G., Fantozzi, P., Muratori, P., Bertolucci, G., Tacchi, A., Villafranca, A., ... & Cortese, S. (2020). Emotional dysregulation and callous unemotional traits as possible predictors of short-term response to methylphenidate monotherapy in drug-naïve youth with ADHD. *Comprehensive psychiatry*, *100*, 152178.

Masi, G., Sesso, G., Pfanner, C., Valente, E., Molesti, A., Placini, F., ... & Milone, A. (2021). An exploratory study of emotional dysregulation dimensions in youth with attention deficit hyperactivity disorder and/or bipolar spectrum disorders. *Frontiers in Psychiatry*, *12*, 619037.

Mauri, M., Grazioli, S., Crippa, A., Bacchetta, A., Pozzoli, U., Bertella, S., ... & Nobile, M. (2020). Hemodynamic and behavioral peculiarities in response to emotional stimuli in children with attention deficit hyperactivity disorder: An fNIRS study. *Journal of Affective Disorders*, *277*, 671-680.

Miranda, A., Soriano, M., Fernández, I., & Meliá, A. (2008). Emotional and behavioral problems in children with attention deficit-hyperactivity disorder: Impact of age and learning disabilities. *Learning Disability Quarterly*, *31*(4), 171-185.

O'Kearney, R., Chng, R. Y., & Salmon, K. (2021). Callous-unemotional features are associated with emotion recognition impairments in young ODD children with low but not high affective arousal. *Child Psychiatry & Human Development*, *52*(5), 869-879.

Özbaran, B., Kalyoncu, T., & Köse, S. (2018). Theory of mind and emotion regulation difficulties in children with ADHD. *Psychiatry research*, *270*, 117-122.

Özyurt, G., Öztürk, Y., & Akay, A. (2017). Relation of emotion regulation and empathy skills with maternal emotion regulation and attachment in children diagnosed with ADHD. *Anatolian Journal of Psychiatry/Anadolu Psikiyatri Dergisi*, *18*(6).

Panteli, M., Constantinou, T., Vrachimi-Souroulla, A., Fanti, K., & Panayiotou, G. (2024). Subjective and autonomic arousal toward emotional stimuli in preadolescents with externalizing problems and the role of explicit and implicit emotion regulation. *Brain Sciences*, *14*(1), 84.

Pate, A., Salmon, K., Fortune, C. A., & O'Kearney, R. (2020). Maternal elaborative language in shared emotion talk with ODD children: Relationship to child emotion competencies. *Child Psychiatry & Human Development*, *51*(2), 254-267.

Perwien, A. R., Kratochvil, C. J., Faries, D., Vaughan, B., Busner, J., Saylor, K. E., ... & Swindle, R. (2008). Emotional expression in children treated with ADHD medication: Development of a new measure. *Journal of Attention Disorders*, *11*(5), 568-579.

Pisano, S., Sesso, G., Senese, V. P., Catone, G., Milone, A., & Masi, G. (2022). The assessment of cyclothymic-hypersensitive temperament in youth with mood disorders and attention deficit hyperactivity disorder. *Journal of Affective Disorders*, *298*, 322-328.

Predescu, E., Sipos, R., Costescu, C. A., Ciocan, A., & Rus, D. I. (2020). Executive functions and emotion regulation in attention-deficit/hyperactivity disorder and borderline intellectual disability. *Journal of clinical medicine*, *9*(4), 986.

Raschle, N. M., Borbás, R., Dimanova, P., Unternaehrer, E., Kohls, G., De Brito, S., ... & Stadler, C. (2025). Losing Control: Prefrontal Emotion Regulation Is Related to Symptom Severity and Predicts Treatment-Related Symptom Change in Adolescent Girls With Conduct Disorder. *Biological Psychiatry: Cognitive Neuroscience and Neuroimaging*, *10*(1), 80-93.

Ros, R., & Graziano, P. A. (2020). A transdiagnostic examination of self-regulation: Comparisons across preschoolers with ASD, ADHD, and typically developing children. *Journal of Clinical Child & Adolescent Psychology*, *49*(4), 493-508.

Rosen, P. J., & Factor, P. I. (2015). Emotional impulsivity and emotional and behavioral difficulties among children with ADHD: An ecological momentary assessment study. *Journal of attention disorders*, *19*(9), 779-793.

Roy, A. K., Bennett, R., Posner, J., Hulvershorn, L., Castellanos, F. X., & Klein, R. G. (2018). Altered intrinsic functional connectivity of the cingulate cortex in children with severe temper outbursts. *Development and psychopathology*, *30*(2), 571-579.
Sanabra, M., Gomez-Hinojosa, T., Grau, N., & Alda, J. A. (2022). Deficient emotional self-regulation and sleep problems in ADHD with and without pharmacological treatment. *Journal of Attention Disorders*, *26*(3), 426-433.

Schoorl, J., van Rijn, S., de Wied, M., Van Goozen, S., & Swaab, H. (2016). Emotion regulation difficulties in boys with oppositional defiant disorder/conduct disorder and the relation with comorbid autism traits and attention deficit traits. *PloS one*, *11*(7), e0159323.

Semrud-Clikeman, M., Walkowiak, J., Wilkinson, A., & Butcher, B. (2010). Executive functioning in children with Asperger syndrome, ADHD-combined type, ADHD-predominately inattentive type, and controls. *Journal of autism and developmental disorders*, *40*(8), 1017-1027.

Seymour, K. E., Chronis-Tuscano, A., Halldorsdottir, T., Stupica, B., Owens, K., & Sacks, T. (2012). Emotion regulation mediates the relationship between ADHD and depressive symptoms in youth. *Journal of abnormal child psychology*, *40*(4), 595-606.

Seymour, K. E., Tang, X., Crocetti, D., Mostofsky, S. H., Miller, M. I., & Rosch, K. S. (2017). Anomalous subcortical morphology in boys, but not girls, with ADHD compared to typically developing controls and correlates with emotion dysregulation. *Psychiatry Research: Neuroimaging*, *261*, 20-28.

Sharifi, A., & Asanjarani, F. (2024). Do ADHD and SLD children differ in executive functions and behavioural problems? A comparative study. *Journal of Research in Special Educational Needs*, *24*(2), 311-323.

Sjöwall, D., & Thorell, L. B. (2019). A critical appraisal of the role of neuropsychological deficits in preschool ADHD. *Child Neuropsychology*, *25*(1), 60-80.

Sobanski, E., Banaschewski, T., Asherson, P., Buitelaar, J., Chen, W., Franke, B., ... & Faraone, S. V. (2010). Emotional lability in children and adolescents with attention deficit/hyperactivity disorder (ADHD): clinical correlates and familial prevalence. *Journal of Child Psychology and Psychiatry*, *51*(8), 915-923.

Spencer, T. J., Faraone, S. V., & Surman, C. B. H. Towards defining deficient emotional self regulation in youth with attention deficit hyperactivity disorder using the CBCL: a controlled study. In *MGH Pediatric Bipolar Disorder Conference*.

Sørensen, L., Plessen, K. J., Nicholas, J., & Lundervold, A. J. (2011). Is behavioral regulation in children with ADHD aggravated by comorbid anxiety disorder?. *Journal of attention disorders*, *15*(1), 56-66.

Taskiran, C., Karaismailoglu, S., Cak Esen, H. T., Tuzun, Z., Erdem, A., Balkanci, Z. D., ... & Cengel Kultur, S. E. (2018). Clinical features and subjective/physiological responses to emotional stimuli in the presence of emotion dysregulation in attention-deficit hyperactivity disorder. *Journal of Clinical and Experimental Neuropsychology*, *40*(4), 389-404.

Tsai, C. J., Lin, H. Y., Tseng, I. W. Y., & Gau, S. S. F. (2021). White matter microstructural integrity correlates of emotion dysregulation in children with ADHD: A diffusion imaging tractography study. *Progress in Neuro-Psychopharmacology and Biological Psychiatry*, *110*, 110325.

Tsai, C. J., Lin, H. Y., Tseng, I. W. Y., & Gau, S. S. F. (2021). Brain voxel-based morphometry correlates of emotion dysregulation in attention-deficit hyperactivity disorder. *Brain Imaging and Behavior*, *15*(3), 1388-1402.

Uran, P., & Kılıç, B. G. (2015). Comparison of neuropsychological performances and behavioral patterns of children with attention deficit hyperactivity disorder and severe mood dysregulation. *European Child & Adolescent Psychiatry*, *24*(1), 21-30

Wu, Z. M., Wang, P., Liu, X. C., Zhou, Q. C., Cao, X. L., Sun, L., ... & Yang, B. R. (2025). Functional and structural connectivity of the subregions of the amygdala in ADHD children with or without ODD. *BMC psychiatry*, *25*(1), 74

Zhang, W., Li, Y., Li, L., Hinshaw, S., & Lin, X. (2023). Vicious cycle of emotion regulation and ODD symptoms among Chinese school-age children with ODD: a random intercept cross-lagged panel model. *Child and Adolescent Psychiatry and Mental Health*, *17*(1), 47.

S4. Race-ethnicity in the included studies.

Study	White	Black	Hispanic	Asian	Pacific	Mixed
Aboelella, 2017						
Akman, 2024						
Alacha, 2024	70.6	22.1	2.9	1.5		2.9
Al-Yagon, 2017						
Becker, 2020	79.6	7.4	4.3	2.5		5.7
Breaux, 2021	82.0	6.0	4.0	4.0		7.0
Bunford, 2015	77.2	11.6				9.4
Bunford, 2017	52.1	47.9				
Bunford, 2018	77.5	16.2				
Chan, 2025	83.2	5.5	3.6	4.7		6.6
Chen, 2022						
Cirey, 2022						
Cleminshaw, 2020	78.4	15.3	5.2	1.1		
Donfrancesco, 2012						
Dunsmore, 2012	77.8	11.1	4.2	2.8		
Dunsmore, 2016	77.5	15.7	4.5	2.2		
Fogleman, 2018	65.4	24.1	3.8			
Gair, 2022	79.6	1.5	2.9	1.5		2.9
Gershy, 2025						
Graziano, 2011	71.0	14.0	11.0			
Graziano, 2013	72.0	15.0	10.0			3.0
Hawkey, 2024	31.0	8.0	21.0			18.0
He, 2023						
Hernandez, 2024	86.7	5.6	81.4	1.9		5.3
Hosiri, 2024						
Hovik, 2017						
Huguet, 2019						
Hulvershorn, 2014	46.0	16.0	37.0	6.0		10.0
Izmir, 2024b						
Jaisle, 2023	66.7	13.9	9.3	0.9		9.3
Jarratt, 2005	83.3	9.5	7.1			
Jensen, 2018						
Jiang, 2016						
Katic, 2013	70.7	19.9	17.4	1.9	0.3	7.3
Kvadsheim, 2020						
Leaberry, 2019	75.0	10.0	7.5			7.5
Lin, 2018						
Linder, 2010						
Liu, 2020						
Liu, 2025						

Mahone, 2007	60.0	36.0			4.0
Maire, 2020					
Marques, 2024					
Masi, 2020	100.0				
Masi, 2021					
Mauri, 2020	100.0				
Miranda, 2008	100.0				
O'Kearney, 2020					
Ozbaran, 2018					
Ozyurt, 2017					
Panteli, 2024					
Pate, 2020					
Perwien, 2008	75.4	12.3	6.2		
Pisano, 2022					
Predescu, 2020					
Raschle, 2025					
Ros, 2020	13.0	3.0	79.0		5.0
Rosen, 2012	59.3	33.3	7.4		
Roy, 2018					
Sanabra, 2022					
Schoorl, 2016	62.0				
Semrud-C., 2010					
Seymour, 2012	40.0				
Seymour, 2017					
Sharifi, 2024					
Sjowall, 2019					
Sobanski, 2010					
Spencer, 2011					
Sørensen, 2010					
Taskiran, 2018					
Tsai, 2021a					
Tsai, 2021b					
Uran, 2015					
Wu, 2025					
Zhang, 2023				100.0	

Note: Empty rows indicate that the study did not provide any data on this variable.

S5. Risk of bias evaluation for the included studies.

Study	1	2	3	4	5	6	7	8	9	10	11	12	13
Aboelella, 2017	Y	Y	NR	NA	N	N	N	NA	Y	NA	Y	N	NA
Akman, 2024	Y	Y	NR	NA	N	N	N	NA	Y	NA	Y	N	NA
Alacha, 2024	Y	Y	N	Y	N	Y	Y	NA	Y	NA	Y	N	Y
Al-Yagon, 2017	Y	Y	NR	NA	N	N	N	NA	Y	NA	Y	N	NA
Becker, 2020	Y	Y	NR	NA	N	N	N	NA	Y	NA	Y	N	NA
Breaux, 2021	Y	Y	NR	NA	N	N	N	NA	Y	NA	Y	N	NA
Bunford, 2015	Y	Y	NR	Y	N	Y	Y	NA	Y	NA	Y	N	NR
Bunford, 2017	Y	Y	NR	Y	N	Y	Y	NA	Y	NA	Y	N	NR
Bunford, 2018	Y	Y	NR	Y	N	Y	Y	NA	Y	NA	Y	N	NR
Chan, 2025	Y	Y	NR	NA	N	Y	Y	NA	Y	NA	Y	N	Y
Chen, 2022	Y	Y	NR	Y	N	Y	Y	NA	Y	NA	Y	N	N
Ciray, 2022	Y	Y	NR	NA	N	N	N	NA	Y	NA	Y	N	NA
Cleminshaw, 2020	Y	y	y	y	Y	N	N	NA	Y	NA	Y	N	NA
Donfrancesco 2012	Y	Y	NR	NA	N	N	N	NA	Y	NA	Y	N	NA
Dunsmore, 2012	Y	Y	NR	Y	N	Y	Y	NA	Y	NA	Y	N	NA
Dunsmore, 2016	Y	Y	Y	Y	N	Y	Y	NA	Y	NA	Y	N	N
Fogleman, 2018	Y	Y	NR	NA	N	N	N	NA	Y	NA	Y	N	NA
Gair 2022	Y	Y	N	NA	Y	Y	Y	NA	Y	NA	Y	N	Y
Gershy, 2025	Y	Y	NR	Y	Y	Y	Y	NA	Y	NA	Y	N	NA
Graziano, 2011	Y	Y	NR	Y	N	N	N	NA	Y	NA	Y	N	NA
Graziano, 2013	Y	Y	NR	Y	N	N	N	NA	Y	NA	Y	N	NA
Hawkey, 2024	Y	Y	Y	Y	Y	Y	Y	NA	Y	NA	Y	N	NA
He, 2023	Y	Y	Y	Y	N	Y	Y	NA	Y	NA	Y	N	Y
Hernandez, 2024	Y	Y	NR	NA	N	N	N	NA	Y	NA	Y	N	NA
Hosiri, 2024	Y	Y	NR	Y	N	N	N	NA	Y	NA	Y	N	NA
Hovik, 2017 (sub)	Y	Y	NR	NA	N	N	N	NA	Y	NA	Y	N	NA
Huguet, 2019	Y	Y	Y	Y	N	Y	Y	NA	Y	NA	Y	N	Y
Hulvershorn, 2014	Y	Y	Y	NA	Y	Y	Y	NA	Y	NA	Y	N	NA
Izmir, 2024b	Y	Y	Y	NA	N	N	N	NA	Y	NA	Y	N	NA
Jaisle, 2023	Y	Y	NR	NA	Y	N	N	NA	Y	NA	Y	N	NA
Jarratt, 2005	Y	Y	NR	NA	N	N	N	NA	Y	NA	Y	N	NA
Jensen, 2018	Y	Y	NR	NA	N	N	N	NA	Y	NA	N	N	NA
Jiang, 2016	Y	Y	NR	NA	N	N	N	NA	Y	NA	Y	N	NA
Katic, 2013	Y	Y	Y	Y	N	Y	Y	NA	Y	NA	Y	N	Y
Kvadsheim, 2020	Y	Y	Y	Y	N	Y	Y	NA	Y	Y	Y	N	NA
Leaberry, 2019	Y	Y	NR	Y	N	N	N	NA	Y	NA	Y	N	NA
Lin, 2018	Y	Y	Y	Y	N	N	N	NA	Y	NA	Y	N	NA
Linder, 2010	Y	Y	NR	NA	N	N	N	NA	Y	NA	Y	N	NA
Liu, 2020	Y	Y	NR	NA	N	N	N	NA	Y	NA	Y	N	NA
Liu, 2025	Y	Y	NR	NA	N	Y	Y	NA	Y	NA	Y	N	NA
Mahone, 2007	Y	N	NR	NA	N	Y	Y	NA	Y	NA	Y	N	NA

Maire, 2020	Y	Y	Y	NA	Y	Y	Y	NA	Y	NA	Y	N	NA
Marques, 2024	Y	Y	NR	NA	Y	N	N	NA	Y	NA	Y	N	NA
Masi, 2020	Y	Y	NR	Y	Y	Y	Y	NA	Y	NA	Y	N	NR
Masi, 2021	Y	Y	NR	NA	N	N	N	NA	Y	NA	Y	N	NA
Mauri, 2020	Y	Y	Y	NA	N	N	N	NA	Y	NA	Y	N	NA
Miranda, 2008	Y	Y	NR	NA	Y	N	N	NA	Y	NA	Y	N	NA
O'Kearney, 2020	Y	Y	NR	NA	N	N	N	NA	Y	NA	Y	N	NA
Ozbaran, 2018	Y	Y	Y	NA	Y	N	N	NA	Y	NA	Y	N	NA
Ozyurt, 2017	Y	Y	NR	NA	N	N	N	NA	Y	NA	Y	N	NA
Panteli, 2024	Y	Y	Y	NA	N	Y	Y	NA	Y	NA	Y	N	NA
Pate, 2020	Y	Y	NR	NA	N	Y	Y	NA	Y	NA	Y	N	NA
Perwien, 2008	Y	Y	NR	NA	N	N	N	NA	Y	NA	Y	N	NA
Pisano, 2022	Y	Y	NR	NA	N	Y	Y	NA	Y	NA	Y	N	NA
Predescu, 2020	Y	Y	NR	NA	N	Y	Y	NA	Y	NA	Y	N	NA
Raschle, 2025	Y	Y	NR	Y	N	N	N	NA	Y	NA	Y	N	N
Ros, 2020	Y	Y	NR	Y	N	Y	Y	NA	Y	NA	Y	N	N
Rosen, 2012	Y	Y	NR	Y	N	Y	Y	NA	Y	NA	Y	N	Y
Roy, 2018	Y	N	Y	NA	Y	Y	Y	NA	Y	NA	Y	N	NA
Sanabra, 2022	Y	Y	NR	NA	N	Y	Y	NA	Y	NA	Y	N	NA
Schoorl, 2016	Y	Y	NR	NA	N	Y	Y	NA	Y	NA	Y	N	NA
Semrud-Clikeman, 2010	Y	N	Y	NA	Y	Y	Y	NA	Y	NA	Y	N	NA
Seymour, 2012	Y	Y	Y	NA	Y	N	N	NA	Y	NA	Y	N	NA
Seymour, 2017	Y	Y	NR	NA	Y	Y	Y	NA	Y	NA	Y	N	NA
Sharifi, 2024	Y	Y	NR	NA	N	N	N	NA	Y	NA	Y	N	NA
Sjowall, 2019	Y	Y	NR	NA	N	Y	Y	NA	Y	NA	Y	N	NA
Sobanski, 2010	Y	N	NR	NA	Y	Y	Y	NA	Y	NA	Y	N	NA
Spencer, 2011	Y	Y	NR	NA	N	Y	Y	NA	Y	NA	Y	N	NA
Sørensen, 2010	Y	Y	N	Y	N	N	N	NA	Y	NA	Y	N	NA
Taskiran, 2018	Y	Y	NR	NA	Y	N	N	NA	Y	NA	Y	N	NA
Tsai, 2021a	Y	Y	NR	NA	N	N	N	NA	Y	NA	Y	N	NA
Tsai, 2021b	Y	Y	NR	NA	N	Y	Y	NA	Y	NA	Y	N	NA
Uran, 2015	Y	Y	NR	NA	Y	Y	Y	NA	Y	NA	Y	N	NA
Wu, 2025	Y	Y	NR	NA	N	Y	Y	NA	Y	NA	Y	N	NA
Zhang, 2023	Y	Y	NR	Y	Y	Y	Y	NA	Y	NA	Y	N	Y

Note: Y = yes, N = No, NA = not applicable, NR = not reported.

Items

1. Was the research question or objective in this paper clearly stated?
2. Was the study population clearly specified and defined?
3. Was the participation rate of eligible persons at least 50%?

4. Were all the subjects selected or recruited from the same or similar populations (including the same time period)? Were inclusion and exclusion criteria for being in the study prespecified and applied uniformly to all participants?

5. Was a sample size justification, power description, or variance and effect estimates provided?

6. For the analyses in this paper, were the exposure(s) of interest measured prior to the outcome(s) being measured?

7. Was the timeframe sufficient so that one could reasonably expect to see an association between exposure and outcome if it existed?

8. For exposures that can vary in amount or level, did the study examine different levels of the exposure as related to the outcome (e.g., categories of exposure, or exposure measured as continuous variable)?

9. Were the exposure measures (independent variables) clearly defined, valid, reliable, and implemented consistently across all study participants?

10. Was the exposure(s) assessed more than once over time?

11. Were the outcome measures (dependent variables) clearly defined, valid, reliable, and implemented consistently across all study participants?

12. Were the outcome assessors blinded to the exposure status of participants?

13. Was loss to follow-up after baseline 20% or less?

14. Were key potential confounding variables measured and adjusted statistically for their impact on the relationship between exposure(s) and outcome(s)?
